# Detrimental Effects of Lipid Peroxidation in Type 2 Diabetes: Exploring the Neutralizing Influence of Antioxidants

**DOI:** 10.3390/antiox11102071

**Published:** 2022-10-20

**Authors:** Samukelisiwe C. Shabalala, Rabia Johnson, Albertus K. Basson, Khanyisani Ziqubu, Nokulunga Hlengwa, Sinenhlanhla X. H. Mthembu, Sihle E. Mabhida, Sithandiwe E. Mazibuko-Mbeje, Sidney Hanser, Ilenia Cirilli, Luca Tiano, Phiwayinkosi V. Dludla

**Affiliations:** 1 Biomedical Research and Innovation Platform, South African Medical Research Council, Tygerberg 7505, South Africa; 2 Department of Biochemistry and Microbiology, University of Zululand, KwaDlangezwa 3880, South Africa; 3 Centre for Cardiometabolic Research Africa (CARMA), Division of Medical Physiology, Stellenbosch University, Tygerberg 7505, South Africa; 4 Department of Biochemistry, Faculty of Natural and Agricultural Sciences, North-West University, Mmabatho 2745, South Africa; 5 Department of Physiology and Environmental Health, University of Limpopo, Sovenga 0727, South Africa; 6 Department of Life and Environmental Sciences, Polytechnic University of Marche, 60131 Ancona, Italy

**Keywords:** lipid peroxidation, oxidative stress, antioxidants, inflammation, type 2 diabetes, metabolic complications

## Abstract

Lipid peroxidation, including its prominent byproducts such as malondialdehyde (MDA) and 4-hydroxy-2-nonenal (4-HNE), has long been linked with worsened metabolic health in patients with type 2 diabetes (T2D). In fact, patients with T2D already display increased levels of lipids in circulation, including low-density lipoprotein-cholesterol and triglycerides, which are easily attacked by reactive oxygen molecules to give rise to lipid peroxidation. This process severely depletes intracellular antioxidants to cause excess generation of oxidative stress. This consequence mainly drives poor glycemic control and metabolic complications that are implicated in the development of cardiovascular disease. The current review explores the pathological relevance of elevated lipid peroxidation products in T2D, especially highlighting their potential role as biomarkers and therapeutic targets in disease severity. In addition, we briefly explain the implication of some prominent antioxidant enzymes/factors involved in the blockade of lipid peroxidation, including termination reactions that involve the effect of antioxidants, such as catalase, coenzyme Q_10_, glutathione peroxidase, and superoxide dismutase, as well as vitamins C and E.

## 1. Introduction

Lipids are certainly the major class of biological molecules, known to play a significant role in various cellular processes such as membrane fusion, fission, endocytosis, protein trafficking, and protein functions [1,2,3]. However, over the past decades, the perception of lipids has dramatically changed due to their essential role in the physiology and pathophysiology of diseases. Lipids such as omega-3 polyunsaturated fatty acids (PUFAs), glycolipids, cholesterol, and its esters are very much susceptible to oxidation, which is a vital mechanism associated with metabolic complications, including the development of cardiovascular diseases [4,5]. Thus, lipid peroxidation is considered as chief pathological mechanism involved in the oxidative damage of cell structures as well as toxicity processes involved in cell death. As such, accumulative literature evidence has shown that lipid peroxidation has an important role in human health and diseases [4,6,7,8]. 

Lipid peroxidation briefly entails an endogenous chain reaction where free radical species such as reactive oxygen species (ROS) cause oxidative degradation of phospholipids resulting in the production of wide a variety of oxidation products [9]. Free radical species for example can attack lipids that contain carbon-carbon bond such as PUFAs in the cellular membrane [10]. The foremost lipid peroxidation byproducts are malondialdehyde (MDA), thiobarbituric acid reactive substances (TBARS), lipid hydroperoxides (LH), and 4-hydroxy-2-Nonenal (4-HNE). Importantly, MDA and 4-HNE are the major aldehydic metabolites of lipid peroxidation that have been widely studied and have been implicated as good biomarkers of lipid peroxidation [11]. These byproducts of lipid peroxidation are involved in oxidative damage of cells, DNA, as well as proteins. Additionally, they can influence signaling pathways involved in oxidative cascade, contributing to the development and severity of many diseases, including the pathophysiology of diseases such as type 2 diabetes (T2D). In fact, lipid peroxidation plays a significant role in the development of atherosclerotic cardiovascular disease, which by far the most cause of death in patients with T2D [12]. Biomarkers of lipid peroxidation including elevated levels of TBARS were significant significantly higher in the plasma of patients with T2D in comparison to healthy subjects [13]. Beyond its implication during the development of T2D, elevated biomarkers of lipid peroxidation have been crucial in highlighting the pathological role of oxidative stress during the pathogenesis of other conditions, including cerebral vascular disease [14,15], obesity-driven cancer [16,17,18], and age-related dementia [19]. Thus, it is essential to recognize antioxidant therapies as a potential strategy to combat lipid peroxidation and oxidative stress during the development of diverse chronic diseases, including T2D [20]. 

Interestingly, available evidence has long established a strong link between poor glycemic control, enhanced generation of lipid oxidation products and deteriorated metabolic health in patients with diabetes [21,22]. Indeed, a clear relationship has been observed between reduced intracellular antioxidant activity and very high lipid peroxide concentrations in diabetic patients [23]. Others have even shown that elevated lipid peroxidation and glutathione peroxidase (GPx) levels, coincide with reduced superoxide (SOD) activity in patients with T2D [24]. Given the rapid rise in cases of diabetes consistent with the evolving landscape of disease etiology [25,26], there is a need to better clarify the pathological coincidence of elevated lipid peroxidation products in patients with T2D. Thus, beyond giving a brief overview of mechanistic insights, the current review explores the pathological relevance of elevated lipid peroxidation products in T2D, especially highlighting their potential role as biomarkers in disease severity. A discussion on the involvement of some prominent antioxidants as terminators of lipid peroxidation processes is included to underscore the potential therapeutic targets to hinder oxidative stress and improve metabolic function in conditions of T2D.

## 2. Prominent Mechanisms Implicated in Generation of Lipid Peroxidation Products

**Figure 1:**
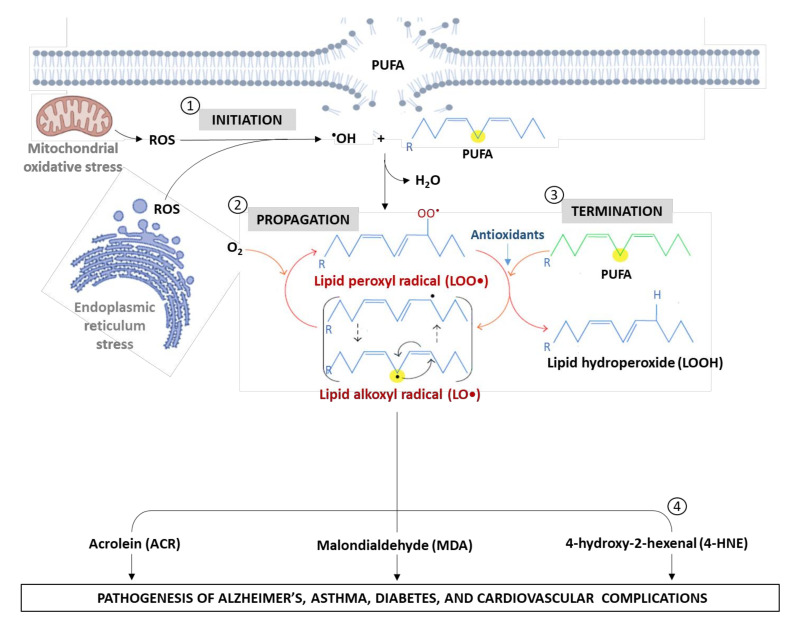
An overview of pathological mechanisms of lipid peroxidation, especially highlighting the formation of lipid aldehydes. Briefly, the different steps, indicate (➀) Initiation, (➁) Propagation (➂) termination, and (➃) formation ACR, MDA and 4-HNE. Abbreviations; PUFA: polyunsaturated fatty acid; ROS: reactive oxygen species; MDA: malondialdehyde; 4-HNE: 4-hydroxy-2-nonenal; •OH: hydroxyl radical; LO•: lipid alkoxyl radical; LOO•: lipid peroxyl radical; LOOH: lipid hydroperoxide. NB: Yellow dot indicated a point where oxidation occurs.

Lipid peroxidation occurs in response to oxidative stress, which is a well-studied pathological process that is characterized by an excessive production of free radical species that cause depletion of intracellular antioxidant reserves [27,28]. As illustrated below, lipid peroxidation describes a miscellaneous pathological mechanism that mainly implicates either non-enzymatic or enzymatic reactions (Figure 1).

### 2.1. Non-Enzymatic Process in Lipid Peroxidation

Non-enzymatic lipid peroxidation process occurs in three distinct steps, which mainly involves the initiation, propagation, and termination. The initiation step is driven by the occurrence of free radicals such as hydroxyl radical (•OH), alkoxyl radical (RO^•^), peroxyl radical (ROO^•^), and HO_2_^•^ that facilitate the production of a high reactive lipid radical (L^•^) through the addition of oxygen molecule and abstraction of a hydrogen atom from a PUFA [29]. Polyunsaturated fatty acids, for that matter, are more susceptible to hydrogen abstraction due to the presence of a double bond that is adjacent to a methylene group making the double bond weaker [30]. The propagation phase is characterized by the addition of molecular oxygen (O_2_) to the lipid radical (L^•^) to generate a lipid peroxyl radical (LOO•) that subsequently attacks other PUFAs. Therefore, the peroxyl radical can abstract a hydrogen atom from several macromolecules such as DNA and proteins, and sort of start a chain reaction [30]. This stage can continue as an uncontrolled self-perpetuating chain reaction, which leads to the amplification of the initial oxidative event and potentially results in the oxidation of all PUFAs in the membrane, forming a short-chain carbonyl derivative, including MDA, 4-HNE, and acrolein. These derivatives subsequently interact and react with nucleic acids and proteins, inducing cellular oxidative damage and contributing to the development of chronic diseases. The last stage in lipid peroxidation known as the termination reaction involves the effect of some prominent antioxidants such as catalase (CAT), Coenzyme Q_10_ (CoQ_10_), glutathione (GSH), superoxide dismutase (SOD), and some vitamins (C and E), which donate a hydrogen atom to the lipid peroxyl radical to form forming a corresponding radical, that reacts with another LOO• to form non-radical products. Subsequently, these antioxidants (discussed in the later section) play an essential role in defusing lipid peroxidation, leading to improved cellular function and amelioration of diabetes-associated complications [21,31]. Numerous studies have reported a reduction in total antioxidant capacity in patients with T2D compared to normoglycemic patients [32,33,34]. 

### 2.2. Enzymatic Process in Lipid Peroxidation

Enzymatic lipid peroxidation is primarily catalyzed by the lipoxygenase’s family, a family of lipid peroxidation consisting of peroxidases, enzymes that oxygenates free and esterified PUFA, generating lipid hydroperoxides than can further decompose to aldehydes such as MDA and 4-HNE. The main enzymes that drive enzymatic process of lipid peroxidation are mainly cytochrome p450, cyclooxygenases, and lipoxygenases [35]. 

## 3. Lipid Peroxidation Biomarkers 

Biomarkers of lipid peroxidation have been established and applied to biological samples [35]. Briefly, the lipid peroxidation process involves the reaction of oxygen and hydrogen radicals with PUFAs, resulting in the formation of LOO^•^ and reactive aldehyde species that are formed as a secondary product of the peroxidation cycle. Thus, due to their stability and highly reactive properties, these byproducts may impair biochemical processes leading to aggravated metabolic function [36,37]. Beyond lipid peroxidation products, free radicals can react with proteins and lead to the formation of advanced glycation end products (AGEs). Like lipid peroxidation, AGES are known to play a vital role in driving oxidative damage by exacerbating complications of diabetes, including retinopathy, nephropathy, neuropathy, and cardiovascular damage [38]. The impact of AGEs in causing cellular damage in patients has been previously discussed [39,40]. Regardless, lipid peroxidation by-products are known to be more deleterious since these conjugates can easily diffuse within the cells and tissue. The major lipid peroxidation biomarkers are MDA, 8-iso-prostaglandin F2α (8-iso-PGF_2_α), F_2_-isoprostane 15(*S*)-8-*iso*-prostaglandin (F_2α_ (15(*S*)-8-*iso*-PGF_2α_), and 4-HNE. These markers are measured in biological samples such as urine, serum, plasma, and saliva as compared to healthy individuals [41,42]. 

### 3.1. Malondialdehyde (MDA) as a Major Biomarker of Lipid Peroxidation

Malondialdehyde is one of the main byproducts of lipid peroxidation associated with the development of several chronic diseases. Malondialdehyde is formed by both enzymatic and non-enzymatic lipid peroxidation of PUFAs such as arachidonic acid and docosahexaenoic acid by cleavage of its double bounds and releasing bis-aldehyde malonaldehyde. This reactive molecule can be used as the primary indicator of overall lipid peroxidation as it is convenient, simple, and low [43]. Under physiological conditions, generally reactive oxygen species including MDA can be formed but easily removed by intracellular antioxidants that are necessary for an efficient biological process. However, elevated levels of MDA-mediated adducts have been associated with inflammation and cellular injury, contributing to the development of cardiovascular and chronic liver diseases [44,45]. Additionally, increased levels of MDA have been associated with the pathogenesis of diabetes mellitus, aging, and other neurodegenerative diseases [46,47]. Malondialdehyde has been reported to inhibit cardiac contractile function through phosphorylation of p38 MAP kinase [48]. Additionally, MDA has been shown to activate the c-Jun N-terminal kinases (JNK)/mitogen-activated protein kinases pathway which mediates both insulin resistance and ß-cell dysfunction [49]. Malondialdehyde levels have been shown to correlate positively with the severity of T2D [50,51,52]. There is a significantly larger number of clinical studies that assessed the effects of different interventions on the modulation of MDA levels in individuals with T2D [53,54,55]. Patients with T2D, at increased risk of coronary artery disease, presented with impaired blood glucose tolerance that was consistent with elevated total-/high-density lipoprotein (HDL)-cholesterol ratio, as well as increased MDA levels and reduced total antioxidant, which was ameliorated with resveratrol treatment [53]. Similarly, impaired glucose and insulin levels were associated with MDA concentrations in patients with T2D and hyperlipidemia, and these effects were ameliorated by with consumption of apple vinegar [55]. With recent findings from a meta-analysis of randomized controlled trials showing that increased blood pressure was related to increased MDA levels in patients with T2D [54]. Such evidence verifies the significance of measuring the levels of MDA to decipher pathological mechanisms of T2D [56]. In addition, a direct measurement of MDA by UV absorption with high-performance liquid chromatography (HPLC) is possible but suffers from inferior sensitivity to MDA-TBA adduct [57]. Overall, MDA has become a routine molecule to signify lipid peroxidation that can be quantified in many different samples, starting from cell organelles to biological fluids (serum, plasma, salivary, and urine) and tissues. 

### 3.2. 4-Hydroxynonenal (HNE) as a Major Biomarker of Lipid Peroxidation

Briefly, 4-hydroxynonenal (HNE) is another quantitatively most important lipid peroxidation byproduct that is highly toxic and has a mutagenic character. As MDA, 4-HNE has been considered as one of the major and the most toxic lipid peroxidation byproduct due to its high capacity to react with multiple macromolecules such as proteins and nucleic acids [58,59]. The reactive aldehyde acts both as a signaling molecule and as cytotoxicity products of lipid peroxidation, mostly generated under the conditions of oxidative stress and can induce long-lasting consequences [60]. Moreover, increased levels of 4-HNE can induce its detrimental effects by in part activating a pro-inflammatory pathway that may be modulated through nuclear factor kappa B (NF-κB), and peroxisome-proliferator-activated receptors (PPAR), leading to the destruction of other cell survival mechanisms that involve autophagy, apoptosis, and necrosis [59,61]. The importance of 4-HNE is mostly emphasized during the pathogenesis of various degenerative and malignant diseases, and for decades it was considered as a cytotoxicity molecule [62]. Due to its high reactivity, 4-HNE is considered as the major contributor to a wide variety of pathological and physiological conditions such as regulating proliferation, differentiation, and apoptosis [63,64]. At lower concentrations, 4-HNE also plays a role in glycemic control by activating peroxisome proliferator-activated receptor δ, thereby increasing insulin secretion by β pancreatic cells [65]. Others have indicated an association between the activation of some PPAR subunits and 4-HNE adducts during the development of some chronic diseases [66,67]. Whereas overexpression of some PPARs such as PPARα can lead to enhanced deposition of glycogen and accelerated apoptosis subsequent to ischemic heart damage in rodents [68]. However, at higher concentrations, 4-HNE triggers various pathways such as apoptosis, autophagy, and necrosis in β pancreatic cells, contributing to the onset and progression of insulin resistance and β pancreatic dysfunction [59]. The most frequently used methods to determine the concentration of 4-HNE are HPLC, and immunoblotting [59,69]. 

## 4. Lipid Peroxidation in Type 2 Diabetes 

Diabetes mellitus is serious metabolic disease that is characterized by abnormally increased glucose levels or uncontrolled hyperglycemia. Defective production of insulin in pancreatic β-cells or impaired insulin action as a result of genetic condition or even secondary to other metabolic complications are considered the key features of diabetes. Indeed, the metabolic anomalies such as “hyperlipidemia” are considered the main consistent with the development of T2D, which the predominant form of diabetes. Currently, about 537 million of the global adult population (20–79 years), a number that is estimated to rise to 783 million by 2045 [25]. This global pandemic of diabetes is largely due to T2D which accounts for more than 95% of people with this condition (WHO, 2021). 

Oxidative stress describes increased production of free radical species, including ROS and reactive nitrogen species), to severely depleted intracellular, a process that drives organ/tissue damage if remains uncontrolled. For example, uncontrolled production of the superoxide radical (O_2_^•–^), mostly generated through impaired actions of the mitochondrial reactive chain and NADPH oxidase, can cause chain activation of other ROS, including •OH and peroxides [70]. In fact, in diabetic conditions, elevated levels of these radicals can easily react with glucose and lipid products, leading to impaired biochemical processes and cell signaling [71]. Lipid peroxidation for that matter defines an oxidative degradation of lipids, which is a destructive feature of oxidative stress leading to damage of cellular membranes, resulting in accelerated apoptosis and cell death [72]. Increased oxidative stress and lipid peroxidation appear to be the detrimental factors associated with insulin resistance, β-cell dysfunction, impaired glucose tolerance, and ultimately T2D [70,73]. Oxidative damage due to increased ROS production is not only involved in the development of T2DM, but also plays a major role in the long-term development of diabetes-related micro- and macro-vascular complications [70]. Even independent of T2D, reduced circulating levels of GSH, GPx, SOD, and vitamin C/E are consistent with lipid peroxidation in patients with cervical cancer [74]. 

**Figure 2:**
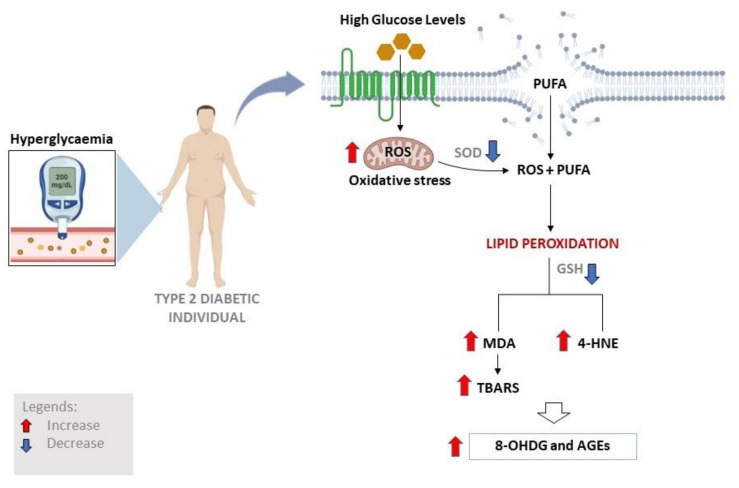
A general overview of the prominent mechanisms and makers of lipid peroxidation such as MDA, 4-HNE, TBARS, that are implicated in type 2 diabetes-related complications. Abbreviations; PUFA: polyunsaturated fatty acid; ROS: reactive oxygen species; SOD: superoxide dismutase; GSH: glutathione; MDA: malondialdehyde; 4-HNE: 4-hydroxy-2-nonenal; TBARS: thiobarbituric acid reactive substances; 8-OHDG; 8-hydroxydeoxyguanosine; AGEs: advanced glycation end products.

Additionally, a recent study reported that MDA levels were significant increase in patients with complications and without complications as compared to their heathy counterparts along with a significant reduction in SOD activity in T2D patients [75]. In agreement, others have shown that MDA levels were significantly elevated in patients with T2D, and this effect was more pronounced in those with microvascular complications when compared to healthy control subjects [47,76]. Apparently, elevated plasma levels of MDA in poorly controlled diabetic patients with dyslipidemia are almost 2.9 times higher when compared to normoglycemic subjects [22]. Few studies have evaluated levels of 4-HNE, another lipid peroxidation biomarker, and showed increased levels of 4-HNE in patients with T2D [77,78]. Furthermore, it has been demonstrated that serum levels of 4-HNE were significantly increased which correlated with disease progression in patients with T2D [12]. Additionally, prominent biomarkers of oxidative stress and lipid peroxidation such as MDA, 4-HNE and ROS levels were significantly increased in preclinical models of diabetes along with a decrease in intracellular antioxidant defenses [79]. In addition, Miwa et al. (2000) suggested that the excessive production of 4-HNE impairs glucose-stimulated insulin secretion in isolated pancreatic β-cells, contributing to the death of pancreatic β-cells in T2D [78]. It is noteworthy that patients with T2D are at increased risk of developing metabolic complications and cardiovascular disease (CVD), especially through the formation of diverse microvascular and macrovascular abnormalities, leading to increased morbidity and mortality [80,81,82]. Apparently, T2D is responsible for at least two-four-fold rise in the incidence of coronary artery disease, which is linked with accelerated heart failure in patients with diabetes [81,82]. Furthermore, biomarkers of cardiometabolic diseases, such as low-density lipoprotein and total cholesterol, in relation with oxidative stress, are much higher in these patients when compared to non-diabetic controls [71,81]. Figure 2 gives a general perspective to highlight some of the pathological consequences of oxidative stress in conditions of hyperglycemia; of which lipid peroxidation is a major component that correlated positively with protein as well as nucleic acid oxidation which is indicated by increased advanced glycation end products (AGEs) and 8-hydroxydeoxyguanosine (8-OHDG), respectively [83,84]. Therefore, decreasing or limiting the formation of lipid peroxidation products could be beneficial in restricting the deleterious effects of oxidative stress in T2D.

## 5. Antioxidants and Their Capacity to Terminate Lipid Peroxidation Products

**Figure 3:**
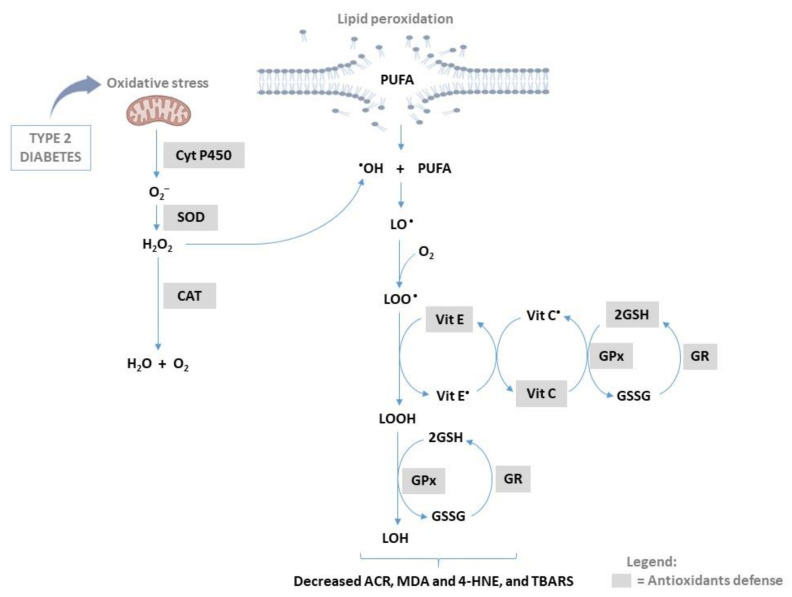
An overview of different antioxidants and their capacity to terminate lipid peroxidation products (especially in conditions of type 2 diabetes). Abbreviations; Cyt P450: cytochrome p450; CAT: catalase; CoQ_10_: Coenzyme Q_10_; PUFA: polyunsaturated fatty acid; ROS: reactive oxygen species; SOD: superoxide dismutase; GSH: glutathione; GSSH: oxidized glutathione; GPx: glutathione peroxidase; GR: glutathione reductase; MDA: malondialdehyde; 4-HNE: 4-hydroxy-2-nonenal; •OH: hydroxyl radical; LO•: lipid alkoxyl radical; LOO•: lipid peroxyl radical; LOOH: lipid hydroperoxides; TBARS: thiobarbituric acid reactive substances; Vit: Vitamin.

For many years, attention has been on the potential protective role of antioxidants and vitamins in ameliorating oxidative stress to diffuse lipid peroxidation-associated damages in T2D (Figure 3). Briefly, in normoglycemia healthy population, lipid peroxidation is suppressed by various antioxidants through decreased production of reactive oxygen, while in T2D, conflicting results have been produced regarding the role of antioxidants and ROS production, experimental studies have suggested that increased lipid peroxidation product are linked with diminished intracellular antioxidant capacity patients with diabetes [24]. Antioxidants can be defined as the group of compounds characterized by their ability to protects cells from the damage of free radicals. In response to enhanced generation of undesired free radical species or ROS, the human body is equipped with intracellular compounds that are known as antioxidants that are necessary for counteracting oxidative stress, thus protecting cellular components against oxidative damage. In fact, the human system contains diverse intracellular antioxidants that can act differently to block the consequences of oxidation stress to maintain cellular function [70,85]. The most common intracellular antioxidants include CAT, CoQ_10_, GSH, and SOD. Briefly, nuclear factor erythroid 2–related factor 2 (Nrf2) is a widely covered transcriptional factor that is widely expressed by all cells and its main function is to regulate genes whose products protect cells against toxicity and oxidative insults [85]. In a physiological state, the cysteine-rich Keap1 (Kelch-like ECH-associated protein 1) protein is responsible for breaking down Nrf2, through the Cul3-mediated ubiquitination process. If not broken down, Nrf2 activation has been highlighted as a significant mechanism in responding against oxidative stress through the modulation of intracellular antioxidants [86,87]. Several studies have shown that increased oxidative stress and lipid peroxidation with a concomitant reduction in antioxidant capacity is associated with decreased pancreatic β-cell mass and cell volume density [35,88]. A recent review has highlighted how cellular activation of Nrf2 is crucial for responding against lipid peroxidation through regulation of different intracellular targets that involve intermediate metabolism, and GSH synthesis/metabolism [87]. Beyond intracellular antioxidants such as CAT, CoQ_10_, GSH, and SOD, extracellular antioxidants such as vitamins C/E have been studied for their potential role in terminating lipid peroxidation in conditions of T2D. Vitamin E supplementation has been potentially linked with the mitigation of oxidative stress and cardiac protection through the activation of Nrf2 in rats [89]. Further highlighting the potential relevance of developing compounds that can activate Nrf2 to block oxidative stress to protect against the development of chronic diseases [90]. Moreover, it has been even suggested that a high dietary intake of antioxidants may lower oxidative stress and delay the development of T2D [91,92,93].

### 5.1. Catalase and Its Effect on Lipid Peroxidation in Type 2 Diabetes

Catalase is a most common enzyme derived from living organisms that protects cells from oxidative damage through elimination of ROS. Through a well-known mechanism, CAT eliminates ROS through conversion of hydrogen peroxide (H_2_O_2_) to H_2_O and O_2._ Hydrogen peroxide is a non-radical ROS that formed through the dismutation of O_2_^•−^ or by the direct reduction in O_2_. Several studies have reported that serum levels of catalase are decreased in patients with T2D [94,95]. Goth et al. (2000) suggested that inherited catalase deficiency is associated with the inability to protect pancreatic β-cells from hydrogen peroxide increasing the risk of T2D. Additionally, another study showed that the onset of T2D appeared to me more than 10 years earlier in catalase deficient patients than the normocatalasemic subjects [96]. However, contradicting results have been produced were some studies reported the compensatory increase in CAT activity in T2D as an indication of increased ROS production [97,98]. 

### 5.2. Coenzyme Q_10_ (CoQ_10_) and Its Effect on Lipid Peroxidation in Type 2 Diabetes

Coenzyme Q_10_, also known as ubiquinone, is a natural occurring lipid-soluble antioxidant found in most body cells. CoQ_10_ plays an essential role in mitochondrial respiratory chain, lipid peroxidation and oxidative stress [99,100]. One of the most important mechanisms through which CoQ_10_ exert its antioxidant properties is the suppression of activity of enzymes involved in the production of ROS [101,102]. In diabetes, several studies have shown that CoQ_10_ levels are decreased in association with excessive production of ROS [103,104]. Additionally, some studies observed a significant reduction in CoQ_10_ levels and a significant higher MDA concentration in patients with T2D compared to normal healthy individuals [105,106]. Other studies, by our group, have suggested that a dietary supplementation with the CoQ_10_ enzyme could reduce the concentration of lipid hydroperoxides, which was consistent with improved metabolic function and reduction conditions of T2D [99,107,108,109].

### 5.3. Glutathione (GSH) and Its Effect on Lipid Peroxidation in Type 2 Diabetes

Glutathione is the most important ubiquitous tripeptide found in most cells which acts as a cofactor in GPx reactions. Glutathione protects cells from oxidative damage by directly scavenging both endogenous and exogenous •OH, nitric oxide, and carbon radicals [110,111]. Briefly, GSH concentrations within the cytosol of many cells is estimated at concentrations ranging from 1–10 mM, with these levels reaching up to 2–10 mM other cell types such as liver and lung cells, as previously reviewed [112]. The predominant mechanism by which GSH removes toxic substances is through conjugation step, leading to its removal of the abducts of the cell [113]. These by-products, which are also consistent with oxidative stress, include hydroperoxides, peroxynitrites, and lipid peroxides [112]. Several studies have shown that increased levels of GSH are associated with the attenuation of neurological damage in experimental models [114]. Some decoctions such as Jiaweibugan have shown the potential against diabetic peripheral neuropathy by protecting neural cells by reducing serum levels of MDA while enhancing concentrations of GSH [115]. Others have demonstrated significantly decreased GSH activity in conditions of T2D when compared to the control group [116,117]. Additionally, it is well known that GSH serves as a cofactor of GPx, a family of enzymes homologous to the selenocysteine that prevent the accumulation of H_2_O_2_ and membrane-associated hydroperoxides [112]. Evidence indicates that a reduction in serum levels of GPx are consistent with impaired blood glucose and oxidative stress in patients with T2D [118]. Additionally, several others have reported a reduced activity of GPx in diabetic patients [119,120], which further confirms the association between T2D-related complications and impaired GSH-strengthening-antioxidative status. 

### 5.4. Superoxide Dismutase (SOD) and Its Effect on Lipid Peroxidation in Type 2 Diabetes

Superoxide dismutase is a metalloenzyme that catalyzes the dismutation of the superoxide radical into molecular oxygen and H_2_O_2_ which is further converted into water by catalase. It has been reported that about 50% of SOD is glycated thus attributing to the reduction in this enzyme [97]. Several studies have suggested a strong association between the SOD activity and T2D [121,122,123]. For example, it has been demonstrated that patients with T2D had a significant lower SOD activity and increased the lipid peroxidation (TBARS) compared to nondiabetics [124]. Supporting this, it has been reported that increased levels of SOD level in significantly reduced in autoimmune inflammatory conditions [125]. In addition, reviewed evidence indicates high concentration of SOD activity in patients with T2D regardless of sex as compared to non-diabetic groups [126]. Whereas loss of SOD has been associated with accelerated oxidative stress in different experimental models, including T2D [127,128]. Additionally, this was evident in a recent study showing that SOD activity was associated with higher MDA levels in patients with T2D [129,130]. 

### 5.5. Vitamins C and E and Their Effect on Lipid Peroxidation in Type 2 Diabetes

Vitamins, particularly vitamin C and E, have been widely reported to protect against free radical-induced cellular damage [131,132]. Briefly, Vitamins C is water-soluble, and it is required for various biological functions [132]. Vitamin C exerts antioxidant properties by acting as an electron donor, preventing molecular compounds from being oxidized. In addition, vitamin C has been reported to attenuate lipid peroxidation in the cellular membranes by scavenging the peroxyl radical, thus leading to improved antioxidant properties of vitamin E [131]. The latter (vitamin E) is classified as a fat-soluble vitamin that is divided into different tocopherols that display enhanced antioxidant properties [133]. A study performed on postmenopausal women with T2D, showed that combined effect of vitamin C and E could significantly improve intracellular antioxidant levels and other metabolic parameters such as lipid profiles and glucose control [134]. Other studies have confirmed that supplementation with both Vitamin C and E has been linked with the attenuation of lipid peroxidation and improved endogenous antioxidant defense systems in patients with diabetes [135,136]. Such effects have been linked with improved CVD-related outcomes in some patients, especially through improvement in blood flow and reduced blood pressure [137,138]. Interestingly, both vitamins C and E have a long history of improving endothelial function, by diffusing lipid peroxidation products and enhancing intracellular antioxidants, especially in a compromised metabolic state [139,140,141].

## 6. Conclusions and Future Perspective

Currently, it is well established that lifestyle derived factors play an important role in driving T2D-related complications. Indeed, overnutrition, coupled by reduced physical activity, may be the paramount factors implicated in worsening of diabetes-related complications [142,143]. In fact, such consequences are strongly linked with the generation of toxic free radicals and oxidative stress, which are linked with increased CVD-risk in patients with diabetes [28,144,145]. As such, lipid peroxidation, as the major consequence of oxidative stress, has been studied for its pathological role in metabolic complications of T2D. At present, common biomarkers of lipid peroxidation such as MDA, TBARS, and 4-HNE are routinely used to detect and estimate the detrimental effects of diabetes [11,21]. Notably, elevation of these biomarkers has been linked with the depletion of intracellular antioxidants (and even increased CVD-risk) in patients with T2D [22,130]. However, controversies have also emerged on the use of only TBARS, especially due to their capacity to react with other components present in biological samples [146,147]. Further highlighting the significance of detecting the combination of these lipid peroxidation products to detect and confirm the severity of oxidative stress within a disease state. Some prominent antioxidants, such as CAT, CoQ_10_, GSH, SOD, and some vitamins (such as C and E), are compromised with the elevation of lipid peroxidation, which usually links with the severity of T2D. On the other hand, increasing research considers antioxidant therapies are considered essential for the alleviation of oxidative stress and better management of T2D [81,148,149,150]. Supplementation with foods rich in antioxidants such as fruits, vegetables, and tea has been linked with lower oxidative stress and enhanced intracellular antioxidants in T2D or related metabolic complications [110,151,152,153]. For example, has already been shown that a diet rich in fruits and vegetables can induce favorable effects in improving serum antioxidant capacity and protect against lipid peroxidation in healthy subjects [154,155]. Preclinical and clinical evidence even suggests that some edible fruits can block lipid peroxidation to enhance antioxidant enzyme activities under conditions of metabolic syndrome or T2D [156,157]. However, as previously discussed [158], although supplementation with antioxidants may be a promising strategy to alleviate oxidative stress and improve metabolic function, much more evidence (especially from clinical trials) is still required to confirm such beneficial effects.
